# Anterior Cervical Pain Syndromes: Defining the Patient Population and Approach to Treatments

**DOI:** 10.7759/cureus.40219

**Published:** 2023-06-10

**Authors:** Jakob L Fischer, Emily A Montgomery, Michael I Orestes

**Affiliations:** 1 Otolaryngology, Walter Reed National Military Medical Center, Bethesda, USA; 2 Surgery, Uniformed Services University of the Health Sciences, Bethesda, USA

**Keywords:** clicking larynx, hyoid bone syndrome, superior laryngeal neuralgia, superior thyroid cornu syndrome, laryngology, throat pain, anterior cervical pain syndrome

## Abstract

Objective

This study aimed to evaluate patients with anterior cervical pain syndromes (ACPSs) by describing patient characteristics, therapeutic interventions, and response to treatments.

Study Design

This is a retrospective observational study.

Methods

Patients treated for diagnoses associated with ACPSs over a seven-year period in one laryngology practice at a tertiary care center were identified and evaluated via a review of clinical and surgical records. Patients identified to have undergone any treatment for ACPSs via medication, trigger-point injections of local anesthetics mixed with steroids, and/or surgical resection of the greater cornu of the hyoid bone and superior cornu of the thyroid cartilage were included. Participants subsequently underwent a medical record review and telephone interview to determine response to treatments.

Results

Twenty-seven patients met the inclusion criteria, including 12 patients (44.4%) with superior laryngeal neuralgia (SLN), seven patients (25.9%) with superior thyroid cornu syndrome (STCS), and eight patients (29.6%) with hyoid bone syndrome (HBS)/clicking larynx syndrome. The most common symptoms were neck/throat pain (27, 100%), globus sensation (20, 74.1%), and dysphagia (20, 74.1%). A total of 24 patients (93.3%) underwent point injections of bupivacaine and dexamethasone. Of these, 12 patients (52.2%) demonstrated a complete response that was permanent in six patients (26.1%). Seven patients (25.9%) underwent surgical intervention, with at least partial improvement noted in six patients (85.7%).

Conclusion

ACPSs constitute a number of complex diagnoses that remain poorly characterized in the literature. The use of point injections of local anesthetics with steroids appears efficacious with surgical options available for those with an incomplete response or return of symptoms.

## Introduction

Anterior cervical pain syndrome (ACPS) is a broad term that encompasses a number of pain syndromes related to the anterior neck that remain under-recognized and undertreated in the otolaryngology patient population. First described by Karlan et al. in 1979, ACPSs describe a number of etiologies of neck pains that are characterized by their paucity of clinical findings. ACPSs include the hyoid bone syndrome (HBS), clicking larynx syndrome, thyroid cartilage syndrome, Eagle syndrome, and carotidynia [1]. Despite encompassing a large number of diagnoses, the literature on ACPSs remains sparse, primarily limited to case series or small-number retrospective analysis. A 2020 paper published by Dewan et al. [2] noted that, at the time of writing, there were only an additional of 17 cases within the literature specifically describing ACPSs. However, most otolaryngologists can attest that this condition is far more commonly seen in the clinical setting [2].

Characteristically, patients with ACPSs demonstrate a paucity of objective clinical abnormalities with most diagnoses made in the absence of any radiographic, endoscopic, or laboratory abnormalities. The hallmark for its diagnosis is pinpoint tenderness within the anterior neck at distinct sites based on their etiologies [1,2]. Given the vague nature of symptoms, patients often experience delays in referral and diagnosis of up to 12 months [3], increasing both patient and physician frustrations.

Multiple papers describe the etiology and various treatment options of conditions considered under the term “ACPS” [1-3], but little comparative literature works exist, to date, relating to presenting symptoms and response to various treatments between etiologies. In this study, we present our experience with 27 patients within an academic otolaryngology practice who underwent treatments for ACPSs over a seven-year period and demonstrate that, independent of specific etiologies, patients tend to favorably respond to trigger-point injections and/or surgical intervention.

## Materials and methods

This study was conducted at Walter Reed National Military Medical Center in Bethesda, Maryland, United States. A database of all surgeries and clinical procedures performed by the senior author (MIO) was reviewed between December 2015 and May 2022. All adult patients (>18 years old) identified to be diagnosed with ACPSs were reviewed. Patients undergoing surgical interventions were identified based on a review of surgical case logs, and patients undergoing injections of local anesthetics and/or steroids were identified based on Current Procedural Terminology (CPT) codes. The review of clinical records based on the chief complaint yielded additional patient records for review. The identified medical records were then reviewed, and the patients were included if they were adult patients diagnosed with ACPSs who underwent evaluations and treatments for this diagnosis. Patients were excluded if they experienced cervical pain secondary to other causes, such as muscle tension dysphonia, traumatic fracture of hyoid or thyroid cartilage, or recurrent laryngeal nerve injury. A total of 41 patients were identified based on these search criteria. Three patients with traumatic hyoid fracture and one patient with a recurrent laryngeal nerve injury were excluded.

Electronic medical records for all included patients were reviewed, including demographics, presenting symptoms, past medical and surgical history, physical examination findings, intra-operative findings, and intervals between treatments. All patients were then contacted telephonically to obtain additional information regarding treatment outcomes. Investigators discussed the nature of the research project with the patients and obtained verbal consent. The patients who were not reachable by phone were subsequently excluded from study participation, resulting in the exclusion of another 10 patients. Based on the chart review and history, the patients were then grouped into various diagnoses within the umbrella of ACPSs. These subgroups were superior thyroid cornu syndrome (STCS), superior laryngeal neuralgia (SLN), and HBS/clicking larynx syndrome diagnosed based on the symptoms and location of discomfort. The specific diagnosis was determined at the time of initial evaluation by the senior author based on the specific location of palpable pain in combination with a careful palpation of the cervical anatomy to evaluate anatomical structures that may be contributing to patient symptomatology. A total of 27 patients were included in the final study cohort.

All data were entered into a de-identified spreadsheet prior to analysis. Descriptive statistics were followed by a univariate analysis of group differences among categorical variables, which included pain location (left, right, bilateral, and referred), dysphagia, pain evoked by different activities (chewing, neck movement, and speaking), voice changes, and perceived sensations (numbness and globus). Given low population numbers within any subgroup for analysis, differences between populations were determined using Fisher’s exact test. A heat map was generated by uploading anonymous matrix data files to Heatmapper (Canadian Institutes of Health Research (CIHR), Genome Alberta), which calculated a pairwise distance matrix using Euclidian measurements [4].

Institutional Review Board approval was obtained from the Department of Clinical Investigations at the Walter Reed National Military Medical Center (approval number: WRNMMC-EDO-2020-0486).

## Results

A total of 27 patients were included in this study. This group included 12 patients (44.4%) with SLN, seven patients (25.9%) with STCS, and eight patients (29.6%) with HBS/clicking larynx syndrome. The average age across all the diagnoses was 48.4 years (range 25-72 years), with 12 patients (44.4%) presenting the disease between the sixth and seventh decades of life. The majority of the patients were male (n=16, 59.3%) and Caucasian (n=15, 55.5%). All patients spoke English as their primary language (Table 1).

**Table 1:** Patient demographics SLN: superior laryngeal neuralgia; HBS: hyoid bone syndrome; STCS: superior thyroid cornu syndrome

Total number of patients	27
Diagnosis	
SLN	12 (44.4%)
HBS/clicking larynx syndrome	8 (29.6%)
STCS	7 (25.9%)
Age	
Mean age	48.4 ± 12.3 (range, 25-72)
18-30 years	3 (11.1%)
31-40 years	5 (18.5%)
41-50 years	4 (14.8%)
51-60 years	12 (44.4%)
>60 years	3 (11.1%)
Gender	
Male	16 (59.3%)
Female	11 (40.7%)
Race	
Caucasian	15 (55.6%)
African American	7 (25.9%)
Asian/Pacific Islander	1 (3.7%)
Unknown/did not disclose	4 (14.8%)
Ethnicity	
Hispanic/Latino	1 (3.7%)
Non-Hispanic/Non-Latino	22 (81.5%)
Unknown	4 (14.8%)

**Figure 1:**
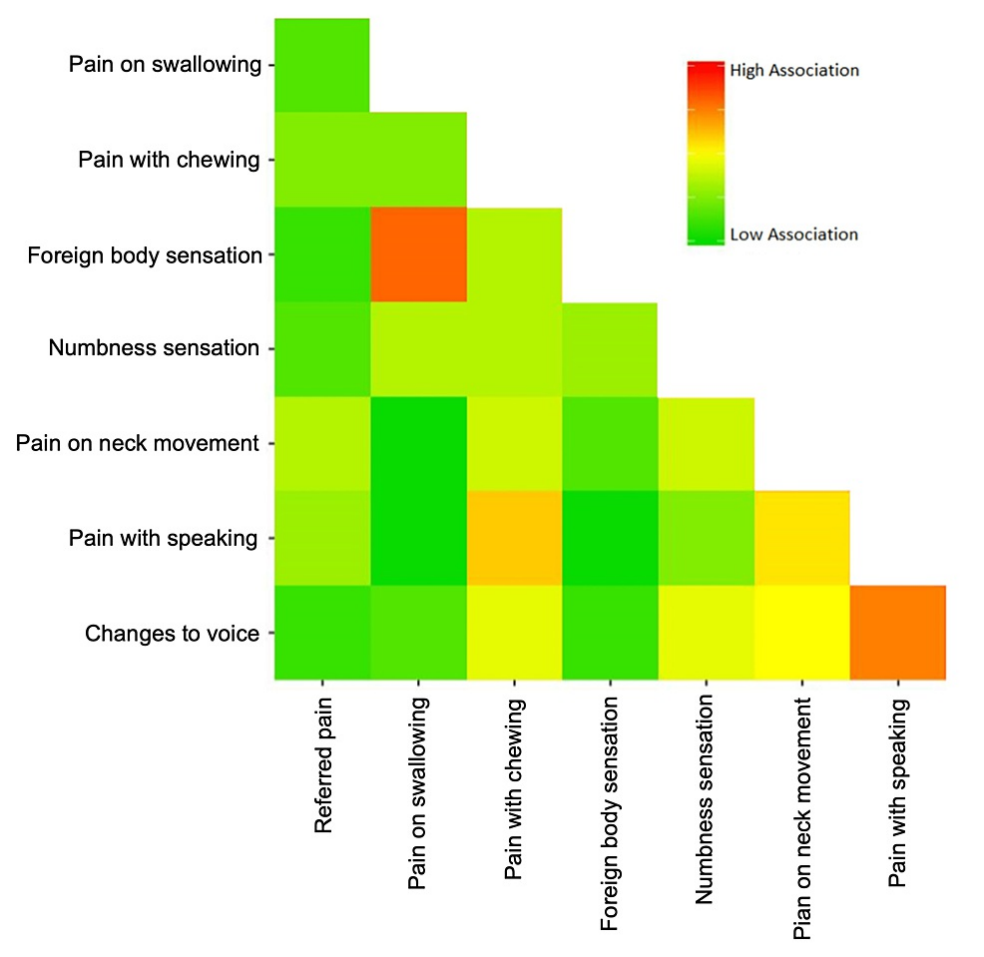
Pairwise frequencies of symptoms experienced by patients with anterior cervical pain syndrome of any etiology

The most common presenting symptom and defining symptom in all the patients was neck/throat pain. Bilateral neck pain was present in five patients (18.5%), which was most commonly associated with HBS/clicking larynx syndrome (n=4, 80%). Seventeen patients (63.0%) experienced referred pain. This was most commonly referred to the ipsilateral mandible/ear (n=9, 52.9%) and lower neck (n=7, 36.8%). The patients most commonly had additional complaints of dysphagia (n=20, 74.1%), foreign body sensation (n=20, 74.1%), and pain on neck movements (n=17, 63.0%). Voice complaints were common (n=15, 55.6%) and included change in the vocal quality (n=9, 60.0%), limitations to pitch (n=3, 20%), and breathiness (n=3, 20%). Pain with chewing was more common in patients diagnosed with STCS (Table 2). Associations with high pairwise frequencies were found between the presence of foreign body sensation and pain with swallowing and between pain with speaking and changes to voice (Figure 1).

**Table 2:** Symptoms in patients presenting with anterolateral cervical pain by diagnosis SLN: superior laryngeal neuralgia; HBS: hyoid bone syndrome; STCS: superior thyroid cornu syndrome

Symptoms	SLN	HBS/clicking larynx syndrome	STCS	Total	P-value
Throat pain (n, %)	12 (100%)	8 (100%)	7 (100%)	27 (100%)	1
Pain laterality: left	8 (66.7%)	3 (37.5%)	5 (71.4%)	16 (59.3%)	-
Pain laterality: right	3 (25%)	1 (12.5%)	2 (28.6%)	6 (22.2%)	-
Pain laterality: bilateral	1 (8.3%)	4 (50%)	0 (0%)	5 (18.5%)	-
Referred pain	8 (66.7%)	4 (50%)	5 (71.4%)	17 (63.0%)	-
Dysphagia	9 (75%)	5 (62.5%)	6 (85.7%)	20 (74.1%)	0.745
Pain with chewing	2 (16.7%)	0 (0%)	4 (57.1%)	6 (22.2%)	0.031
Pain with neck movements	8 (66.7%)	5 (62.5%)	4 (57.1%)	17 (63.0%)	0.334
Pain with speaking	5 (41.7%)	3 (37.5%)	3 (42.9%)	11 (41.0%)	1
Foreign body sensation	8 (66.7%)	5 (62.5%)	7 (100%)	20 (74.1%)	0.238
Numbness sensation	2 (16.7%)	0 (0%)	0 (0%)	2 (7.4%)	0.487
Voice changes	9 (75%)	3 (37.5%)	3 (42.9%)	15 (55.6%)	0.227

In total, 24 patients (88.9%) underwent point injection of a 50:50 mixture of 0.25% bupivacaine and 10 mg/mL dexamethasone. In each patient, the area of point tenderness was directly palpated, and the patients provided verbal confirmation on the location of pain. A 1.0 mL mixture of anesthetic and steroid was infiltrated directly at the area of tenderness and massaged into the tissues. Twelve of the 24 patients (50%) undergoing point injection demonstrated a complete response (including both permanent and temporary complete responses) to the injection that was permanent in six of the patients (i.e., 50% of those that had a complete response had a complete permanent response). In total, 19 of the 24 patients (79.2%) experienced at least a partial response to the injection alone (Table 3). The patients with an incomplete or temporary response reported a high variability in the duration of symptom reduction. Four patients experienced only transient relief, four patients experienced relief for one week, and the remaining four patients experienced relief for one to four months. Eight patients (33.3%) elected to undergo a second trigger-point injection, and three patients (12.5%) underwent three or more injections. There was no difference in the completeness of response or duration of relief based on an underlying diagnosis. The majority of the patients receiving at least two injections (n=5, 62.5%) were patients with SLN. The duration and degree of symptom resolution was similar between the initial and subsequent injections in all the patients.

**Table 3:** Response to treatments in patients undergoing trigger-point injection and surgical resection of the greater cornu of the hyoid and superior thyroid cornu cartilage *Two patients underwent subsequent contralateral procedure with resolution of symptoms; **patient underwent additional resection of styloid process. SLN: superior laryngeal neuralgia; HBS: hyoid bone syndrome; STCS: superior thyroid cornu syndrome

Trigger-point injection (n=24)	SLN (n=10)	HBS/clicking larynx syndrome (n=7)	STCS (n=7)	Total	P-value
Complete response, permanent	3 (30%)	1 (14.3%)	2 (28.6%)	6 (25%)	
Complete response, temporary	3 (30%)	2 (28.6%)	1 (14.3%)	6 (25%)	
Partial response	3 (30%)	2 (28.6%)	2 (28.6%)	7 (29.2%)	
At least partial response	9 (90%)	5 (71.4%)	5 (71.4%)	19 (79.2%)	0.550
No response	1 (10%)	2 (26.6%)	2 (26.6%)	5 (18.5%)	
Surgical resection (n=7)					
Complete response	2	-	-	3 (42.9%)	
Partial response	1	3*	-	2 (28.6%)	
No response	-	1**	-	1 (14.3%)	

Surgical intervention was offered to the patients with partial relief of symptoms with injection. Of the 27 patients included in our overall study, seven patients (25.9%) eventually underwent surgical intervention. Of these seven patients, five had undergone prior trigger-point injection and experienced transient relief of symptoms lasting between one day and one week after injection. Two patients elected to defer trigger-point injection and proceed directly to surgery. All the patients, except for one noted to have an elongated styloid process, underwent ipsilateral resection of the greater cornu of the hyoid bone and superior cornu of the thyroid cartilage. Six patients (85.7%) experienced at least a partial relief in symptoms, with two patients (28.6%) experiencing permanent complete relief. Two patients diagnosed with HBS/clicking larynx syndrome noted persistent symptoms after surgery that improved with contralateral resection. There was no difference in outcomes based on a pre-operative diagnosis when comparing the efficacy of anesthetic/steroid injection or surgical outcome.

## Discussion

**Figure 2:**
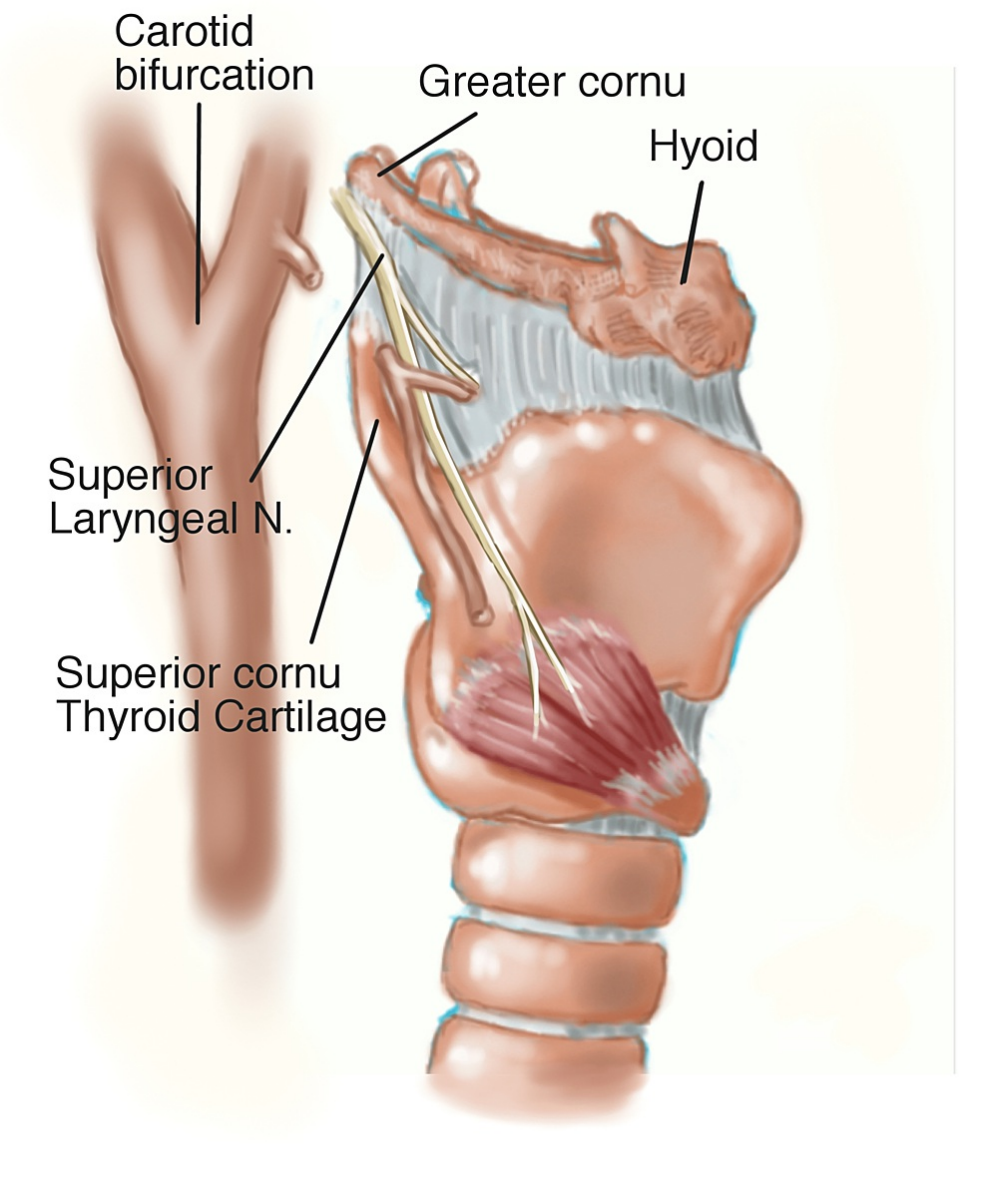
Lateral view of the larynx depicting anatomical structures implicated in etiologies associated with ACPSs, including the greater cornu of the hyoid bone, superior cornu of the thyroid cartilage, superior laryngeal nerve, and bifurcation of the carotid artery associated with carotidynia Image Credit:  Dr. Gary Wind, MD, FACS ACPSs: anterior cervical pain syndromes

ACPSs encompass several anatomic and neurologic abnormalities that may cause anterior neck pain. Most patients had no discernible factors that precipitated the onset of symptoms, and symptoms among patients varied substantially. The defining characteristic of these patients was the presence of cervical tenderness that was often localized to a single trigger point location in the anterior neck. Despite pinpoint tenderness being a defining symptom, definitive diagnosis remains difficult in the patients, possibly secondary to the proximity and complex anatomy adjacent to the areas of point tenderness (Figure 2).

In our patient population, we have demonstrated that, independent of etiology, patients with physical exam findings indicative of ACPSs generally respond well to injection of a mixture of local anesthetics and steroids. We found that over a quarter of patients will experience permanent, complete relief with trigger-point injection, and up to 70% of patients will receive at least some benefit from a trial of anesthetic/steroid injection. The patients who noted only transient relief of symptoms tended toward preferring surgical intervention, which we found to completely resolve symptoms in two patients who otherwise had only partial responses to injection.

Our findings are consistent with existing literature regarding patients with ACPSs and diagnoses under the ACPS umbrella. Dewan et al. [2] reported a 50% rate of symptom resolution in ACPSs with trigger-point infiltrations of lidocaine and triamcinolone separately. In patients with HBS, multiple retrospective studies have demonstrated the efficacy of trigger-point injection. Rubin et al. demonstrated that 84% (n=45) of patients with HBS had a significant benefit with trigger-point injection of the greater cornu of the hyoid bones with triamcinolone. This study did not include local anesthetics in their injection and noted that relief was often delayed by up to a week. Follow-up was also limited, and further delayed benefit from the steroid injection may not have been captured [5]. Additional retrospective reviews have demonstrated response rates between 66% and 74% [6,7].

Kunachak et al. performed a retrospective analysis examining the efficacy of triamcinolone injections in 51 patients with ACPSs secondary to HBS, STCS, cricoid pain, and carotidynia. They noted success rates after a single injection of 36.8% (n=19) in HBS, 50% (n=16) in greater thyroid cornu syndrome, 62.5% (n=8) in cricoid cartilage syndrome, and lower rates in other adjacent pain syndromes. In their population, the single-injection success rate was 33%, which improved to 100% in the fourth injection [8]. In our patient population, successive injections yielded results similar to their initial injection, with no patients having a duration of improvement longer than the initial injection.

Surgery was typically reserved for patients who noted only a transient partial or complete resolution of symptoms with injections. Two patients elected to proceed to surgical intervention up front in lieu of injection. Surgery was effective in achieving at least a partial resolution of symptoms in 85.6% of patients, with 28.6% demonstrating complete resolution that was permanent. Surgical resection of the thyroid cartilage and hyoid bone has shown promising early results in ACPSs with another small series demonstrating a resolution of symptoms in 100% of patients (n=5). Other studies examining diagnoses associated with ACPSs have had promising surgical results with success rates of 100% (n=11) with the clicking larynx syndrome [9], 90% (n=18) with HBS [10], and 75% (n=8) with STCS [11].

Three of our surgical patients were diagnosed with SLN, all of which responded to surgery. The literature regarding surgical intervention for SLN remains particularly limited as most published data do not include surgical options. One small study (n=2) attempted surgical sectioning of the superior laryngeal nerve at the thyrohyoid membrane, with both patients demonstrating initial improvement in pain symptoms and subsequent recurrence two and four years later [12]. Another small study of patients with refractory SLN have demonstrated successful pain relief for at least 14 months without evidence of recurrent symptoms [13]. This is consistent with multiple claims supporting SLN resection for intractable cases [14,15]. A proposed alternative therapy in the treatment of SLN is gamma knife radiosurgery (GKRS). A pilot study of three patients who underwent GKRS demonstrated resolution of pain at 30-32 months of follow-up in all three patients [16]. It appears that the resection of the ipsilateral superior cornu of the thyroid cartilage and greater cornu of the hyoid bone may be a viable option in patients with ACPSs of all etiologies, although further study will be necessary to determine the efficacy in patients with SLN.

We propose that management begins with a close physical examination to include attempts at targeted palpation of the neck to evaluate for pinpoint tenderness. It is worthwhile to examine any patient with vocal complaints with flexible laryngoscopy to evaluate for any other pathologies that may be contributing to neck pain, such as muscle tension dysphonia [17]. In patients with findings consistent with ACPSs, we recommend trigger-point injection with a mixture of lidocaine or bupivacaine and steroids, such as triamcinolone. We have found that patients with ACPSs tended to have immediate relief with injection of local anesthetics while steroids may provide longer term efficacy. In patients who experience only temporary relief of symptoms, they may then be offered resection of the ipsilateral hyoid bone and greater cornu of the thyroid cartilage. In patients with HBS/clicking larynx syndrome, consideration could be made to perform bilateral surgery, although we have elected to treat the more symptomatic side first and reserve contralateral surgery for those that failed to resolve.

For patients who may be poor surgical candidates or apprehensive of in-clinic procedures, non-steroidal anti-inflammatory medications (NSAIDs) may be an option. A retrospective review of 38 patients with HBS demonstrated symptomatic relief in 66% of patients that significantly increased to 91% in patients with symptoms of less than six weeks duration [18]. Smaller studies have demonstrated improvement in symptoms related to the use of carbamazepine although these remain limited by their sample size (Brownstone et al. reported successful carbamazepine treatment in one case report patient, while Schmidt et al. reported on a patient who experienced resolution of pain with compliance but return of mild pain possible due to irregular intakes of the medication) [19,20]. Lee et al. demonstrated symptomatic relief in 68% of patients (n=28) treated with gabapentin [21]. Amitriptyline is an additional medication that has been frequently used in the treatment of neuropathic pain. There is limited evidence for its use in the treatment of ACPSs, with a single randomized controlled trial demonstrating subjective improvement in 67% of patients (n=9) with chronic laryngopharyngeal neuropathy [22]. A 2015 Cochrane review examining the efficacy of amitriptyline in neuropathic pain of all etiologies found that it may be efficacious in a minority of patient populations [23].

There are multiple limitations to this study. There are no generally applicable codes within the electronic medical records that are specific to ACPSs or any subset of diagnosis; as such, patients were identified based on chief complaints and procedural and surgical codes. This automatically excludes any patients that may have been evaluated for ACPSs and undergone medical treatment alone or declined injection, but it may indicate that patients included in the patient population experienced prolonged course of the disease and/or disease that was non-responsive to oral therapies. This study is also limited to one surgeon’s experience and the limitations inherent to retrospective observational studies without a comparison group. There is a risk of recall bias in this patient population, as the patients were interviewed in some cases years after the treatment. This study was limited to adult patients and ACPSs have not been substantially described in a pediatric population. Further studies would be necessary to evaluate the clinical characteristics and response to treatments in this population.

## Conclusions

ACPSs describe several diagnoses with limited literature directly comparing diagnostic and therapeutic options. Patients with ACPSs can present with several different symptoms but typically present with neck/throat pain, foreign body sensation, and dysphagia, although several other symptoms may be present. Independent of etiology, findings show that trigger-point injections with local anesthetics and steroids are efficacious to provide at least a partial or temporary response in the majority of the patient population. However, the option remains for surgical resection of the superior thyroid cornu and greater cornu of the hyoid in patients who experience return of symptoms or incomplete response.
